# The calcium-activated chloride channel-associated protein rCLCA2 is expressed throughout rat epidermis, facilitates apoptosis and is downmodulated by UVB

**DOI:** 10.1007/s00418-021-01962-5

**Published:** 2021-01-23

**Authors:** L. Hämäläinen, G. Bart, P. Takabe, L. Rauhala, A. Deen, S. Pasonen-Seppänen, E. Kärkkäinen, R. Kärnä, T. Kumlin, M. I. Tammi, R. H. Tammi

**Affiliations:** 1 Institute of Biomedicine/Anatomy, University of Eastern Finland, P.O. Box 1627, N70211 Kuopio, Finland; 2 Department of Environmental and Biological Sciences, University of Eastern Finland, P.O. Box 1627, N70211 Kuopio, Finland

**Keywords:** rCLCA2, REK, Epidermis, UVR, Apoptosis

## Abstract

The rodent chloride channel regulatory proteins mCLCA2 and its porcine and human homologues pCLCA2 and hCLCA2 are expressed in keratinocytes but their localization and significance in the epidermis have remained elusive. hCLCA2 regulates cancer cell migration, invasion and apoptosis, and its loss predicts poor prognosis in many tumors. Here, we studied the influences of epidermal maturation and UV-irradiation (UVR) on rCLCA2 (previous rCLCA5) expression in cultured rat epidermal keratinocytes (REK) and correlated the results with *mCLCA2* expression in mouse skin in vivo. Furthermore, we explored the influence of rCLCA2 silencing on UVR-induced apoptosis. r*Clca2 *mRNA was strongly expressed in REK cells, and its level in organotypic cultures remained unchanged during the epidermal maturation process from a single cell layer to fully differentiated, stratified cultures. Immunostaining confirmed its uniform localization throughout the epidermal layers in REK cultures and in rat skin. A single dose of UVR modestly downregulated r*Clca2 *expression in organotypic REK cultures. The immunohistochemical staining showed that CLCA2 localized in basal and spinous layers also in mouse skin, and repeated UVR induced its partial loss. Interestingly, silencing of rCLCA2 reduced the number of apoptotic cells induced by UVR, suggesting that by facilitating apoptosis, CLCA2 may protect keratinocytes against the risk of malignancy posed by UVB-induced corrupt DNA.

**Supplementary Information:**

The online version contains supplementary material available at 10.1007/s00418-021-01962-5.

## Introduction

Skin and particularly its most superficial part, epidermis, form the barrier around our body, protecting from harmful insults. To fulfill this task, the epidermal cells continuously proliferate and differentiate, regenerating the barrier in a tightly regulated manner. When an insult damages the barrier, mechanisms are needed to correct the situation as soon as possible. Among the multitude of mechanisms involved in this response, chloride channels including Clc-2 and -3, and CFTR have recently gained interest (Chen et al. 2016; Dong et al. 2015; Guo et al. 2016). They may be involved in the regulation of keratinocyte migration, proliferation, and differentiation (Dong et al. 2015; Guo et al. 2016; Pan et al. 2015) and tumor suppression (Zhang et al. 2013). Chloride channel accessory proteins (CLCA) may regulate the activity of chloride channels (reviewed in Patel et al. 2009; Walia et al. 2012; Yurtsever et al. 2012). hCLCA2 and its porcine (pCLCA2) and mouse (mCLCA2, former mCLCA5) homologues are expressed by epidermal keratinocytes (Braun et al. 2010; Connon et al. 2004; Plog et al. 2012). Also, the other CLCA family members mCLCA3A1 (former mCLCA1), mCLCA3A2 (former mCLCA2*)* and rCLCA5 (former rCLCA2) are expressed in epidermis (Bart et al. 2014; Elble and Pauli 2001; Leverkoehne et al. 2002; Yamazaki et al. 2013)

CLCA proteins are transmembrane molecules proteolytically cleaved into a ~ 35-kDa C-terminal fragment associated with the plasma membrane, and a ~ 90-kDa N-terminal, secreted fragment (reviewed in (Patel et al. 2009)). Via regulating chloride channels, the CLCA proteins can modulate cell proliferation and apoptosis (reviewed in Patel et al. 2009). They also contain integrin-binding domains via which they can promote cell adhesion and control cell migration and invasion (Abdel-Ghany et al. 2003; Sasaki et al. 2012; Walia et al. 2012). More recently, it has been shown that CLCA proteins regulate intracellular Ca-signaling by complexing with the ER and cell surface Ca channels STIM1 and Orai1. This possibly enables control of diverse cellular processes such as differentiation (Sharma et al. 2018).

The loss of *hCLCA2* correlates with the development of many tumors (Bustin et al. 2001; Elble and Pauli 2001; Gruber and Pauli 1999a; Qiang et al. 2018; Riker et al. 2008; Shinmura et al. 2014; Tanikawa et al. 2012; Walia et al. 2009; Zhao et al. 2011), yet the role of CLCA proteins in the epidermis is unsettled. hCLCA2 may protect keratinocytes from hyperosmotic stress (Seltmann et al. 2018). rCLCA5 appears to be associated with epidermal differentiation (Bart et al. 2014; Yamazaki et al. 2013). mCLCA2 and pCLCA2 proteins have been localized in the uppermost vital cell layers in the epidermis and suggested to be involved in differentiation (Braun et al. 2010; Plog et al. 2012). However, when mouse keratinocytes are induced to differentiate with high calcium medium, no change in *mClca2* mRNA and protein expression is observed (Hiromatsu et al. 2015). Because of the CLCA family differences between human, mouse and rat, it is possible that the functions of CLCA2 orthologues in the epidermis are not equal in different species. Supporting this hypothesis, Seltman and colleagues showed that *mClca3A2* (former *mClca2*), rather than *mClca2*, shows similar responses to dehydration and hyperosmotic stress as *hCLCA2* (Seltmann et al. 2018). Unlike the homologs in the human, pig and mouse, the expression or functions of rCLCA2 in the epidermis have not been studied before.

Here, we show that rCLCA2 has relatively high and stable mRNA and protein expression level throughout the different stages of epidermal maturation. In addition, we show that rCLCA2 facilitates UV-induced apoptosis in keratinocytes, and it may also itself be a UVR target gene.

## Materials and methods

### Keratinocyte culture

REKs (Baden and Kubilus 1983) were maintained as monolayer cultures in MEM (Thermo Fisher Scientific/Gibco, Waltham, MA) containing 10% FBS (HyClone, Thermo), 4 mM L-glutamine, 50 μg/ml streptomycin and 50 U/ml penicillin. For immunostainings, the cells were grown on chamber slides (Nalge Nunc, Thermo Fisher Scientific Inc., Waltham, MA).

### Maturation assay

The maturation assay was performed as described before (Hämäläinen et al. 2018). REKs were seeded on polycarbonate culture inserts in 12-well plates (0.4 µm pore size, Thermo Fisher Scientific/Gibco) in MEM containing 10% FBS. After lifting to the air–liquid interface on day 3, the cultures were incubated in DMEM (Invitrogen/Gibco) with 10% FBS, L-glutamine, and antibiotics.

### UVB irradiation

For the UVB and sham treatments, the REK organotypic cultures were transferred to Dulbecco’s PBS (Euroclone). A portable UV-lamp (UVM-57; UVP, Upland, CA) emitting mid-range UV at a nominal wavelength of 302 nm was used for the UVB treatments as described previously (Bart et al. 2014). The peak radiation of the lamp at 312–313 nm was verified by spectroradiometry as described previously (Bart et al. 2014).

### Immunostainings

The UVR-treated mouse samples were fixed in neutral buffered formalin, while the other specimens were fixed in Histochoice (Amresco, Solon, OH). Rehydrated paraffin sections were sequentially incubated in 1% H_2_O_2_ and 1% bovine serum albumin in 0.1 M phosphate buffer, pH 7.4. An overnight incubation with the anti-CLCA2 antibody (M-60, Santa Cruz Biotechnology, Santa Cruz, CA; 1:200–1:2000) was followed by incubations with biotinylated anti-rabbit IgG (1:500) and avidin–biotin peroxidase (both from Vector Laboratories, Burlingame, CA). The color was developed with 0.05% 3,3′-diaminobenzidine (Sigma-Aldrich) containing 0.03% H_2_O_2_. The nuclei were stained with Mayer’s hematoxylin. Imaging was performed using Zeiss AxioImager M2 (Carl Zeiss AG, Oberkochen, Germany, Plan-Neofluar 40 × 0.75 NA objective) with AxioCam MRc camera.

For colocalization analysis, the anti-CLCA2 antibody (1:500) was mixed with anti-TGN46 antibody (1:1000, PA5-23,068, Invitrogen). At the secondary step, Texas Red (TR)- and FITC-labeled anti-rabbit and mouse IgG were mixed (1:500, Vector). To label endocytic vesicles, TR-labelled transferrin (25 µg/ml, T2875, Molecular Probes) was added to the cultures for 30 min. After fixation, the cells were stained for CLCA2 using FITC-labelled anti-rabbit IgG. Confocal images were taken with a 40 × NA 1.3 oil objective on a Zeiss Axio Observer inverted microscope with a Zeiss LSM 700 confocal module.

**Fig. 1:**
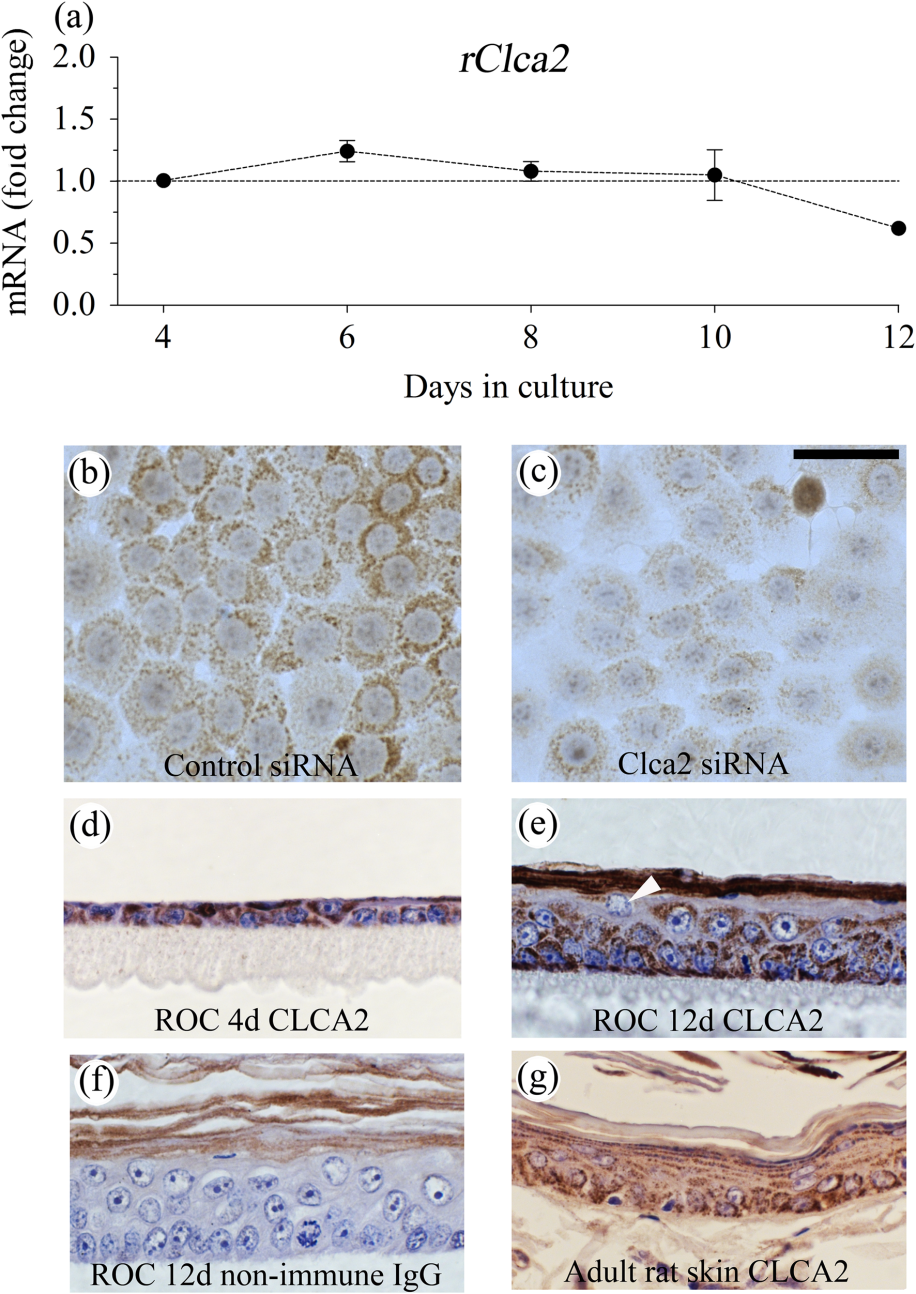
Rat epidermal keratinocytes express CLCA2. REK cells were grown in organotypic cultures (ROC) where they stratify and differentiate (**a**, **d**, **e**, **f**). **a** Samples were collected for qRT-PCR at the indicated time points (3 independent experiments, each with duplicate cultures). **d**, **e**, **f** Immunostaining with an anti-CLCA2 antibody or non-immune IgG at day 4 (**d**) and day 12 (**e**, **f**) (3 independent experiments). **b**, **c** REK cells grown as monolayers were transfected with control (**b**) and *rClca2*-specific siRNA (**c**) and stained for rCLCA2. The experiment was repeated 4 times. In all experiments, CLCA2 immunostaining intensity was lower in cultures treated with *Clca2* siRNA as compared to those with control siRNA. **g** Adult rat skin skin of 3 animals was stained for rCLCA2

The r/mCLCA2 antibody is directed against amino acids 601–660 of mCLCA2, which show 93% sequence homology between these species. This site is localized at the N-terminal side of the postulated cleavage site of the full-length pro-form of the molecule (Patel et al. 2009). The specificity of the antibody was controlled: (1) Using non-immune rabbit IgG instead of the primary antibody (Fig. 1f). (2) By silencing *rClca2* expression in REK cells with specific siRNAs (Fig. 1b). (3) By staining mouse kidney and rat submandibular and parotid glands. The observed staining patterns did not correspond to those published for mCLCA3A1 and mCLCA3A2 in the kidney (Roussa et al. 2010) or rCLCA5 in the salivary glands (Yamazaki et al. 2005) (data not shown) excluding the cross reactivity of the anti-m/rCLCA2 antibody with these CLCAs.

### Mouse skin exposed to chronic UVR

Archived specimens of UVR-treated mouse skin were used for CLCA2 immunostainings (Kumlin et al. 1998). The back skin of the mice was exposed to UVR three times a week for 10.5 months with a lamp simulating solar spectrum (280–400 nm) (Philips HP3136, Eindhoven, The Netherlands). Each single UVR dose corresponded to one human minimum erythema dose (Kumlin et al. 1998).

CLCA2 staining pattern was evaluated using a 40 × objective. Each consecutive field was classified as either regular, uniformly stained or irregular, containing focal weakly stained or negative area among the intensely stained cells. The morphological type of the epidermis (normal/mild hyperplasia, moderate/strong hyperplasia, dysplasia) and the presence of SCC were also recorded. Some of the specimens contained areas of 2 or 3 epidermal morphological types and/or SCC. Irregular staining was classified into four levels scored from 1 to 4. Score 1 was given when 0–25% of the fields contained focally reduced staining; while scores 2, 3 and 4 were assigned to samples containing reduced staining in 26–50%, 51–75% and 76–100% of fields, respectively. Samples with poor tissue preservation or size too small for representative analysis were omitted from the analyses.

### SiRNA transfections, apoptosis, cytotoxicity and migration assays

RNAiMAX was used to transfect REK cells with control siRNA (Origene, Rockville, Maryland, USA) or r*Clca2* siRNA (Eurogentech, Liège, Belgium). 30 nM final concentrations were used for the immunostainings, apoptosis and cytotoxicity assays and 50 nM for the migration assays. 24 h after transfection, the cells were fixed for the immunostainings. For the caspase 3/7 activation assay and for the cytotoxicity assay, the cells were trypsinized and seeded in 96-well plates from Greiner (Bio-One, Frickenhausen, Germany) and Perkin Elmer (Waltham, MA), respectively. For migration assays, the cells were seeded on 24-well plates (Greiner).

A day after plating, the media in 96-well plates were replaced with PBS and the cells were exposed to 10 mJ/cm^2^ of UVB, or sham treated. Thereafter, fresh culture medium was added. To study caspase 3/7 activation, IncuCyte® Caspase-3/7 Red -dye (Sartorius, Ann Arbor, MI) was added to the medium at 0.25 μM final concentration, and the cells were incubated for 24 h in IncuCyte S3 live cell imaging system (Sartorius). Imaging of the cells were done hourly. Data analysis was performed using IncuCyte Software (Sartorius), normalizing the caspase 3/7 signals to cell confluence.

For the cytotoxicity assay (Promega, Madison, WI), 24 h after the UVB and sham exposures, AAF-Glo™ substrate (Promega) was added to the wells. After 15-min incubation at room temperature, the luminescence was measured using Luminoskan™ Ascent (Thermo Fisher Scientific) combined with Ascent Software. To measure the total cytotoxicity, lysis reagent containing digitonin was added, and the incubation and luminescence scan steps were repeated. As a positive control, some unexposed wells received 200 μM H_2_O_2_, and for a background control, we had medium-only (no cells) wells.

In the migration assay, cultures incubated for 24 h after the plating were wounded with a pipet tip. The cultures were photographed immediately after the scratching and 6 h later using an Olympus CK2 inverted phase contrast microscope (Olympus Optical Co. Ltd., Tokyo, Japan, 4 × objective) with a Nikon Digital Sight DS-L1 camera system. The cell-free areas were measured using NIH ImageJ software (National Institutes of Health, USA; http://imagej.nih.gov/ij).

### Extraction of total RNA

The epidermis was lyzed, and RNA was extracted with TRI Reagent® (Molecular Research Center, Cincinnati, OH, U.S.A.). The purity and quantity of total RNA were measured with a NanoDrop ND-1000 spectrophotometer (Thermo Fisher Scientific).

### cDNA synthesis and quantitative RT-PCR

1 µg of total RNA was used as a template for cDNA synthesis with Verso™ cDNA synthesis Kit (Thermo Fisher Scientific). qRT-PCR analyses were done on Stratagene Mx3000P cycler (Agilent Technologies, Inc.) using FastStart Universal SYBR Green Master with ROX (Roche). Gene-specific primer pairs and cycling conditions are listed in Supplemental Table 1. Fold changes were calculated as described (Livak and Schmittgen 2001).

### Statistical methods

qRT-PCR data were analyzed with one-way ANOVA with Dunnett’s Multiple Comparisons Test (for organotypic time series) or two-way ANOVA with Bonferroni post-test (for UVB-treated samples) using GraphPad Prism 5.03 for Windows (GraphPad Software, La Jolla, CA). The apoptosis data were analyzed using a linear mixed model with the Sidak pairwise comparisons between UVB-exposed control and *Clca2*-siRNA-transfected cultures with SPSS Statistics 27 (IBM®). Evaluation of the CLCA2 staining patterns in mouse specimens was done using Mann–Whitney *U* test and the migration data using paired *t* test.

## Results and discussion

### rClca2 expression is not changed during epidermal maturation

According to the qRT-PCR data, *Clca2* is expressed in cultured rat keratinocytes (REK) at a relatively high level (mean ΔCt-value: 6.7 and 7.0 in monolayer and in organotypic cultures, respectively). r*Clca2* mRNA expression level remained rather stable throughout the maturation period in the organotypic cultures, originating from a single cell layer (day 4) up to fully stratified and cornified epidermis (day 12) (Fig. 1a).

**Fig. 2:**
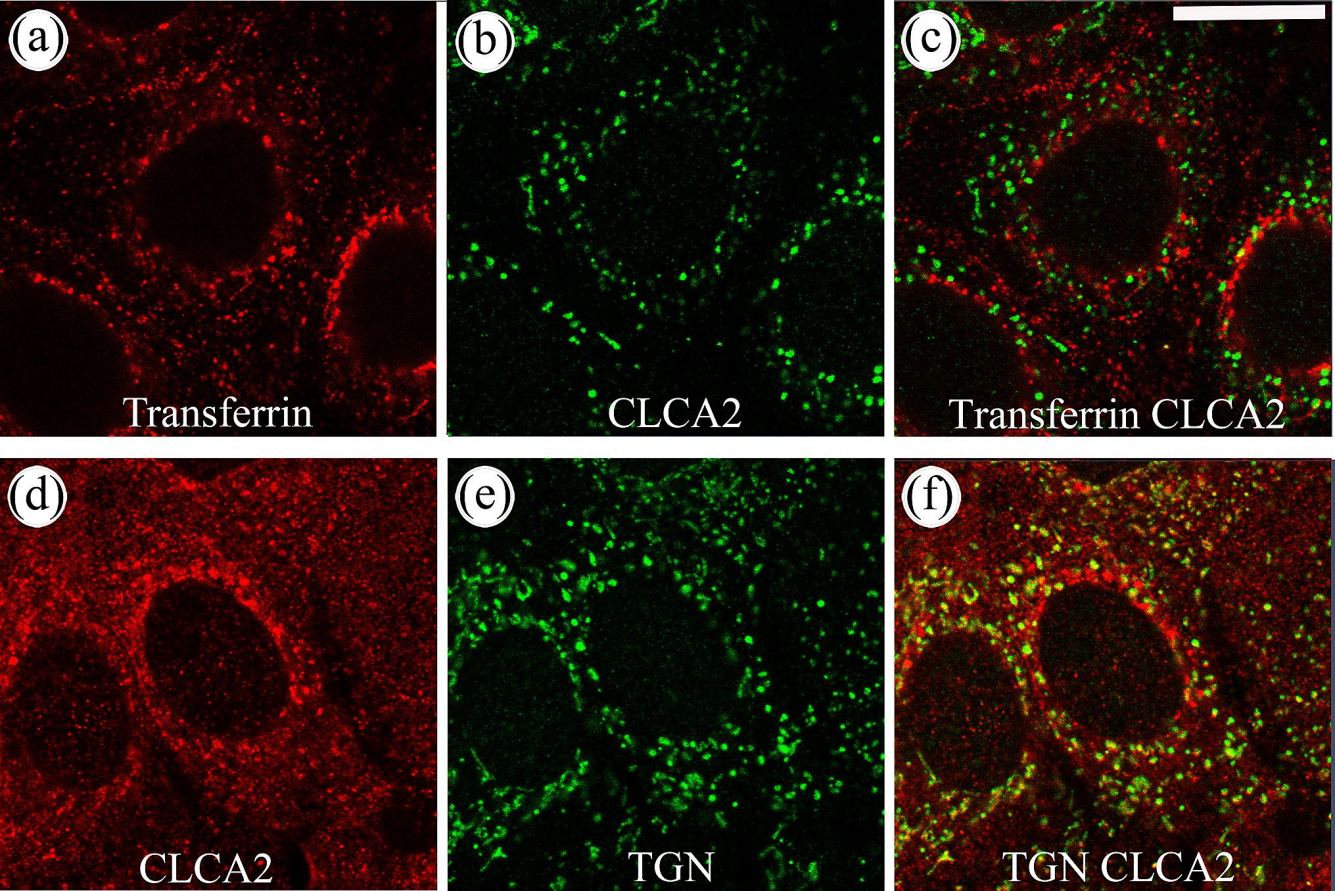
CLCA2 shows a partial colocalization with trans-golgi network (TGN). **a**–**c** Monolayer REK cell cultures were incubated with Texas Red-transferrin for 30 min, fixed and stained with an anti-rCLCA2 antibody. In panels **d**–**f** REK cells were stained for TGN (green) and CLCA2 (red). The data represent 3 individual experiments, each performed with duplicates. Magnification bar represents 50 µm

The anti-m/rCLCA2 antibody gave a strong signal in REK monolayers with a grossly granular or vesicular intracellular pattern (Fig. 1b). Knockdown of *rClca2* with specific siRNAs resulted in a clear reduction of the rCLCA2 immunostaining (Fig. 1b, c). The efficacy of the mRNA knockdown was about 80% (Supplemental Fig. 1). Immunofluorescence staining with confocal microscopy confirmed that the CLCA2 antibody stained intracellular granular and vesicular structures close to the nucleus and further off in the cytoplasm but failed to show any plasma membrane staining (Fig. 2). In dual stainings with the endosomal marker transferrin, it showed just an occasional colocalization (Fig. 2a–c); while, it colocalized more substantially with an antibody recognizing the trans-Golgi network (TGN, Fig. 2d–f). However, only a part of the TGN vesicles were CLCA2 positive.

In REK organotypic cultures, the r/mCLCA2 antibody stained keratinocytes throughout their different stages of maturation (Fig. 1d, e). As in the monolayers, the staining was intracellular, while plasma membranes were not stained. All vital cell layers were positive although the intensity was somewhat lower in the granular layer compared to the basal and spinous cells (Fig. 1e, arrow). The distribution of the CLCA2 immunostaining was similar in skin (Fig. 1g) and other stratified epithelial tissues of the rat, such as tongue and stomach (data not shown). Non-immune rabbit IgG used as a negative control gave no signal in the vital parts of the epidermis, while stratum corneum showed non-specific staining (Fig. 1f).

In its equal expression in all vital cell layers, *Clca2* differs from *rClca5,* the expression of which clearly increases with epidermal maturation. In fully differentiated REK cultures and in rat epidermis, the full-length *rClca5* localizes in upper spinous and granular layers; while, its truncated splicing isoform is mainly located in the undifferentiated basal cell layer (Bart et al. 2014; Yamazaki et al. 2013).

Similar to *rClca2,* its mouse orthologue *mClca2* is expressed in the epidermis and other stratified epithelia at relatively high copy numbers (Braun et al. 2010). No change in *mClca2* mRNA or protein expression has been observed in mouse keratinocytes induced to differentiate with the switch to high calcium medium (Hiromatsu et al. 2015), suggesting that its expression is similar to the rat orthologue and not dependent on the stage of differentiation. In line with this notion, the m/rCLCA2 antibody used here stained with equal intensity the basal and suprabasal cell layers in mouse epidermis (Fig. 4b). However, another study using a different antibody found mCLCA2 only in the granular cells (Braun et al. 2010). Epitopes used in immunization (in N-terminal, C-terminal, or membrane-associated parts) and their sensitivity to fixation could explain the differences in the findings. Moreover, the site of processing of the protein and the distribution of its fragments may also differ between cell types and species. Thus, the processing of mCLCA2 occurs in Golgi (Braun et al. 2010), whereas that of hCLCA2 takes place at cell surface, and the human N-terminal fragment remains associated with the C-terminal part at plasma membrane (Elble et al. 2006).

The immunostaining of the porcine orthologue pCLCA2 localizes in the spinous and granular cells (Plog et al. 2012) while the hCLCA2 was originally reported to localize in the basement membrane zone (Connon et al. 2004). However, a newer human antibody (Atlas antibodies, #HPA047192) appears to stain both basal and spinous cell layers in the skin, esophagus and cervix, which suggests a distribution similar to that in rat and mouse.

Although hCLCA2 is expressed also in some simple epithelia like breast epithelium (Gruber and Pauli 1999b), it is especially strongly expressed in stratified epithelia like tongue, esophagus, cervix uteri, and vagina (Beckley et al. 2004; Braun et al. 2010), and therefore considered a marker of stratification. Indeed, in tissue culture models of human and chicken corneal epithelium, CLCA2 expression is induced when undifferentiated embryonic and stem cells in monolayer are committed to stratification (Connon et al. 2006). The REKs in our work were isolated from newborn rat, already committed to the epidermal lineage (Baden and Kubilus 1983), and, therefore, show high *Clca2* expression also in monolayer.

### rCLCA2 has a proapoptotic function in epidermis

**Fig. 3:**
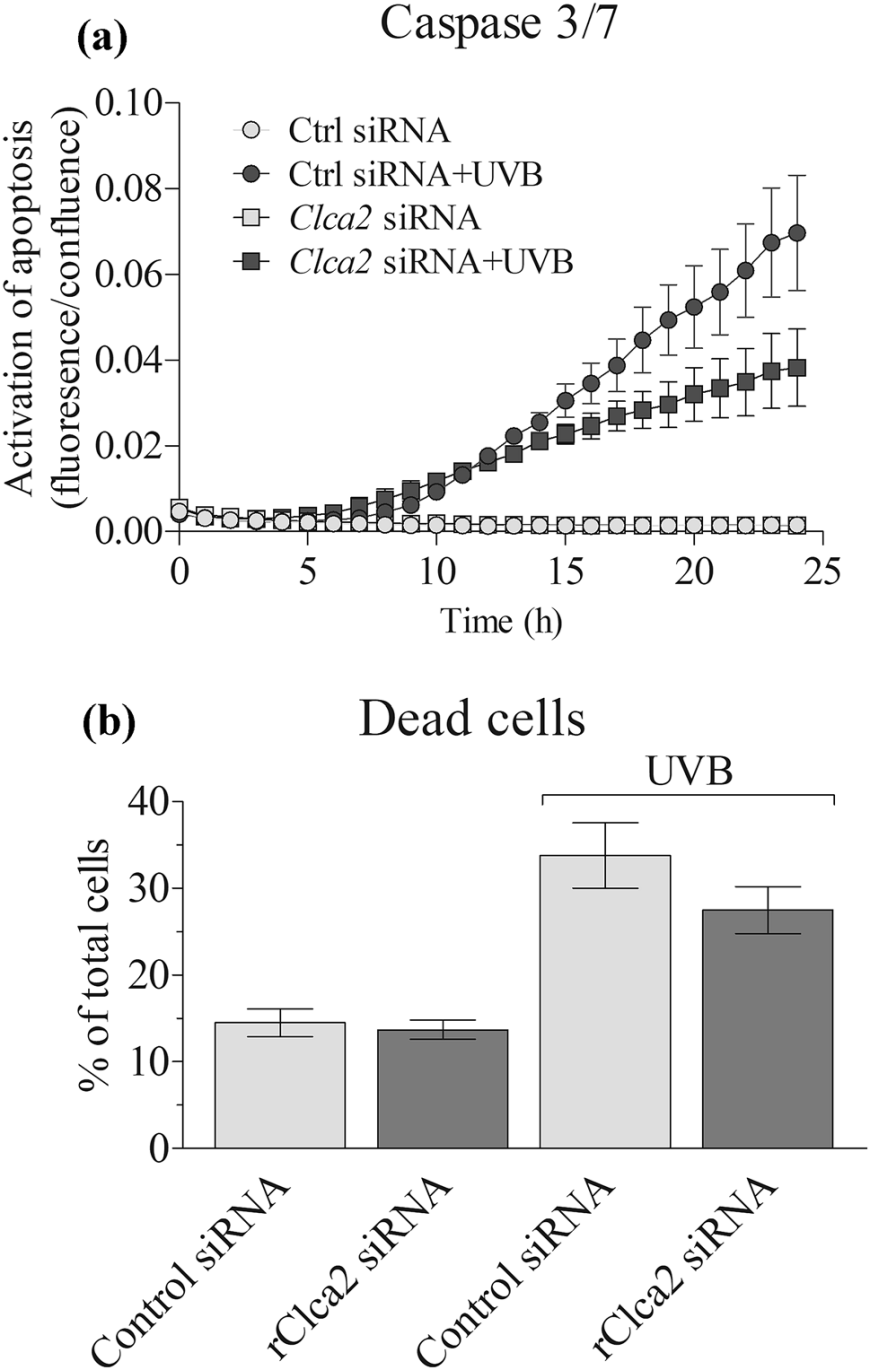
Clca2 silencing reduces UVR-induced cell death in REKs. CLCA2 expression was silenced in REK cells in monolayer cultures. 2 days after the silencing, the cells were either exposed to UVB (10 mJ/cm^2^) or sham exposed. **a** The cultures were analyzed for caspase 3/7 expression using Incucyte imaging system (3 independent experiments, each with 4 replicate wells). The difference between Cntr siRNA + UVB and *Clca2* siRNA + UVB was statistically significant (*p* < 0.001, linear mixed model). **b** The membrane integrity of the cultures was analyzed using Cytotoxicity assay (2 independent experiments, each with 3 replicate wells) as described in “Materials and methods”

Human and mouse homologs of rCLCA2 have been associated with apoptosis in breast epithelial cells (Beckley et al. 2004; Walia et al. 2009). To see if rCLCA2 has a similar function in rat keratinocytes, we silenced *rClca2* expression and examined its influence on UVB-induced apoptosis by analyzing the appearance of cells positive for caspase 3/7. Under basal culture conditions the numbers of caspase 3/7 -positive cells were low and *rClca2* silencing showed no effect on it (Fig. 3a). A 10 mJ/cm^2^ dose of UVB caused a clear increase in caspase 3/7 positive cells after 24 h in cultures treated with control siRNA (Fig. 3a). This rate of apoptosis was reduced by 50% in *Clca2-*silenced cultures (Fig. 3a). The CytoTox-assay, which measures the damage of cellular membranes, showed a similar trend (Fig. 3b). The findings indicate that rCLCA2 has a proapoptotic role in keratinocytes subject to UVR-induced stress. This can be important for removing cells harboring damaged DNA. In line with the present data, overexpression of mCLCA2 and hCLCA2 enhances apoptosis in mammary epithelial cells (Beckley et al. 2004; Walia et al. 2009) and its silencing makes MCF10 cells more resistant to cytotoxic drugs (Walia et al. 2009). However, in human keratinocytes, *Clca2* silencing failed to significantly reduce UVB-induced cell death (Seltmann et al. 2018). On the other hand, keratinocytes exposed to hyperosmotic stress responded to hCLCA2 silencing with apoptosis (Seltmann et al. 2018). The data suggest that CLCA2 may exert opposite effects on different cell death signaling pathways activated by different stressors.

Human CLCA2 has been reported to inhibit breast and nasopharyngeal cancer cell motility (Qiang et al. 2018; Sasaki et al. 2012; Walia et al. 2012) and its knockdown leads to increased migration and invasion (Connon et al. 2005; Qiang et al. 2018). In the rat, however, siRNA silencing of *rClca2* did not significantly influence REK cell migration in a scratch wound assay (Supplemental Fig. 2).

### UVR downmodulates rCLCA2 expression

**Fig. 4:**
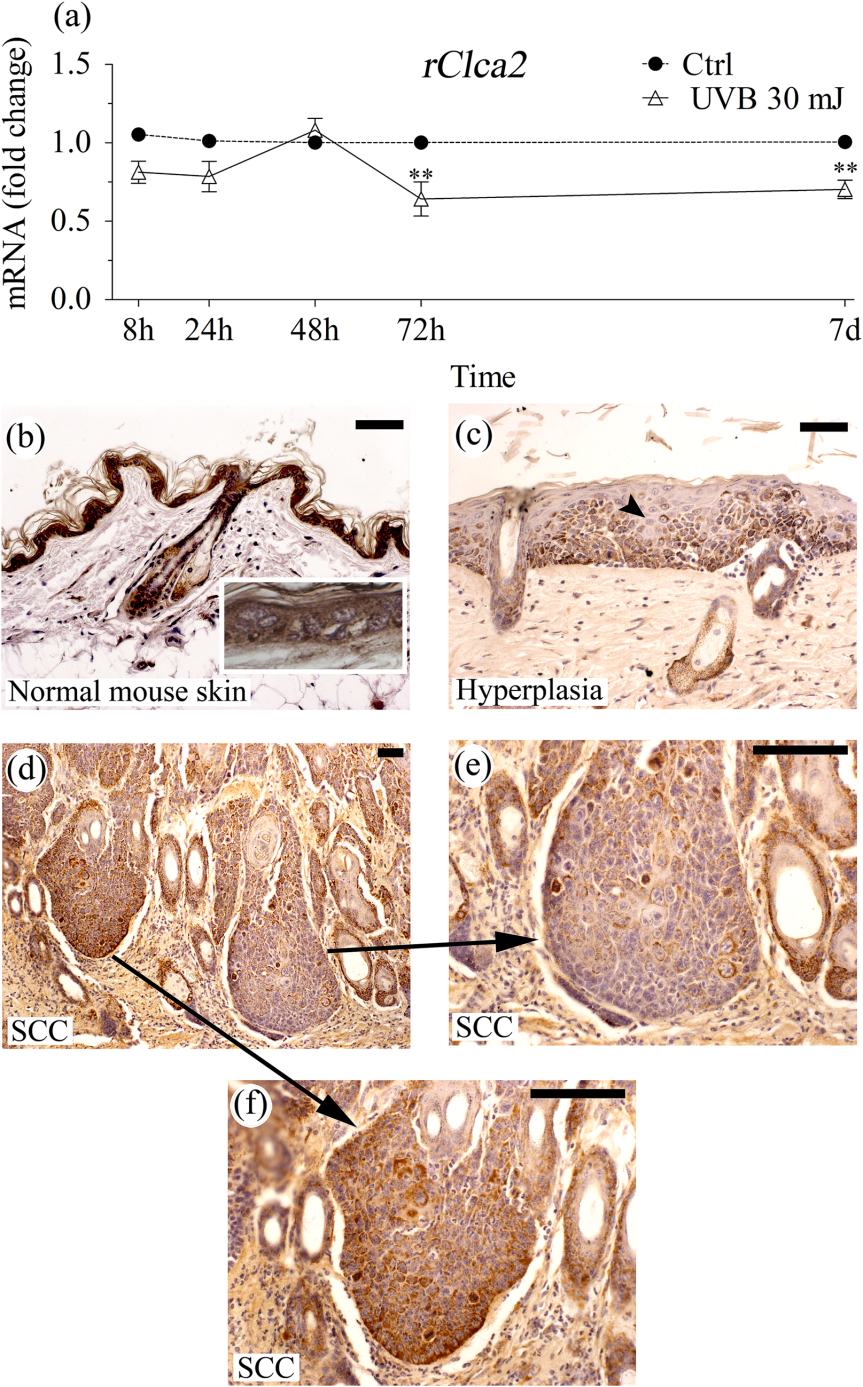
Clca2 is downmodulated by UVR. **a** REK organotypic cultures (9 days) were either sham-treated or UVB-exposed, and samples were collected at the indicated time points and analyzed for r*Clca2* mRNA. The Ct-value of the sham-treated culture was set as 1 at each time point and the mRNA level of the UV-exposed culture was compared to it. The data represent means and SE from 3 individual experiments, each performed with duplicate cultures. The statistical differences were tested using 2-way ANOVA with Bonferroni’s Post Hoc test. **p < 0.01. **b**–**f** Histological sections from 14 sham (**b**) or 14 UVR (**c**–**f**)-exposed mouse back skins were stained with anti-CLCA2 antibody. **b** The sham-exposed skin with normal epidermal morphology shows intense, regular immunostaining in the epidermis. Both basal and the suprabasal cells are CLCA2 positive (**b**, inset) as well as the hair follicles and sebaceous glands. **c** A UVR-exposed specimen with strong epidermal hyperplasia showing irregular, focally reduced CLCA2 staining (arrowhead). **d**–**f** A UVR-exposed specimen with SCC. **d** Overview, **e** an area with focally reduced CLCA2 immunostaining, **f** an area with intense regular staining. Similar irregular pattern was observed in all 5 SCCs and 8 hyperplastic areas found in the specimens. Magnification bars 50 µm

Since CLCA2 is involved in the regulation of apoptosis when keratinocytes are stressed with UVR, as shown above, we wanted to study whether the expression of CLCA2 itself is affected by UVR. The importance of this question is stressed by the fact that UVR is the main risk factor for skin cancers. A single 30 mJ/cm^2^ dose of UVB, effective but not strongly destructive (Bart et al. 2014), caused ~ 20% down-modulation of r*Clca2* mRNA in organotypic REK cultures 8 h after the exposure (Fig. 4a). Despite this relatively modest original effect, the inhibition of UVB on *Clca2* expression was still detected 7 days after the irradiation (Fig. 4a). A change in the general immunostaining intensity of CLCA2 could not be distinguished after the single UVB exposure (not shown). Due to the high sensitivity of the REK cells to the UVB irradiation, we could not test if raising the UVB dose or repeated doses would have resulted in a more substantial decrease in *rClca2* expression.

The effects of UVR on the expression of *Clca* family members vary among different species. For example, in human keratinocytes,* hCLCA2* does not respond to a single UVB exposure (Seltmann et al. 2018); while in mouse embryonic fibroblasts, UVB upregulates mCLCA2 expression in a p53-dependent way (Sasaki et al. 2012). In rat keratinocytes, a strong downregulation of *rClca5* was found (Bart et al. 2014). UVR induced an irregular signal also in mouse epidermis and SCC stained with an anti-rCLCA5 antibody, likely detecting mCLCA3A2 (former mCLCA2) (Bart, Hämäläinen et al. 2014; Hiromatsu et al. 2015), which is strongly expressed in mouse epidermis (Elble and Pauli 2001; Seltmann et al. 2018).

UVR activates several signaling routes, which differ between the experimental models and the UVR doses used (Syed et al. 2013). p53 activation by UVB was not seen in the REK organotypic model (Bart et al. 2014), a result that corresponds to that in human skin in vivo (Enk et al. 2004). Among the known negative regulators of CLCA2 expression is the transcription factor Fra1 (Zhao et al. 2014), whose mRNA expression and protein stability is increased by UVB in keratinocytes (Hopper et al. 2009; Jung et al. 2016; Silvers and Bowden 2002). Accordingly, several other Fra1 targets, such as TGFβ and keratins 10 and 16 (Benhadou et al. 2020; Zolotarenko et al. 2018), are affected by UVB in REK organotypic cultures (Bart et al. 2014; Rauhala et al. 2015). The long-lasting influence of UVB on CLCA2 expression may involve epigenetic effects through methylation or microRNA, shown to regulate CLCA2 expression in breast, colorectal and prostate cancers (Li et al. 2004; Porretti et al. 2018; Tanikawa et al. 2012).

### CLCA2 in carcinogenesis

The suppression of apoptosis following *Clca2* downregulation in UVR-exposed keratinocytes can be physiologically significant since it can contribute to the maintenance of skin integrity by preventing excessive cell death. However, at the same time, it can also facilitate survival of damaged cells and thereby favors carcinogenesis. To get insight into the long-term influences of UV stress on CLCA2 expression, we immunostained CLCA2 in skin samples collected from mice exposed to chronic UVR. The normal epidermis in sham-exposed specimens was intensely stained with the anti-CLCA2 antibody (Fig. 4b). In the UVR-treated specimens the CLCA2 staining was also mostly intense but contained spots of focally reduced CLCA2 signal (Fig. 4c–e). When the frequency of this irregular staining pattern was scored in an 1–4 scale, all 14 specimens in the sham-exposed group got the lowest score (1); while in the UVR-treated group, only 4 out of 14 specimens were assigned into this category (Supplementary Table 2, *p* < 0.001). The irregular staining in the UVR-treated group correlated with the epidermal morphology. Areas with strong epidermal hyperplasia or dysplasia showed more irregular staining than the epidermal areas where UVR caused no visible change (Fig. 4c, Supplementary Table 2). Irregular staining was found also throughout the UVR-induced SCCs (*n* = 5), but the cells in the invasive front showed no preference for the reduced CLCA2 signal (Fig. 4d–f, Supplementary Table 2).

The loss of hCLCA2 in colorectal, breast and ovarian cancers and melanoma correlates with increased tumorigenicity (Bustin et al. 2001; Li et al. 2004; Riker et al. 2008; Zhao et al. 2011), and the expression is low also in lung adenocarcinoma (Shinmura et al. 2014), indicating a role as a tumor suppressor. Our data suggesting that mCLCA2 is decreased in the UVR-induced SCC are in line with these findings. Moreover, among the lung SCCs with a generally strong signal, there were cases showing reduced or totally lost CLCA2 immunostaining and this pattern correlated with high tumor grade and poor prognosis among female patients (Shinmura et al. 2014).

### Conclusions

rCLCA2 is strongly expressed throughout the vital epidermis with equal expression levels in proliferative and differentiating cells. Silencing of rCLCA2 reduces UVB-induced apoptosis and cytotoxicity, indicating that rCLCA2 has a proapoptotic role in the epidermis, important in preventing survival of damaged cells. It appears also to be a UVR target gene, being downmodulated by irradiation in the epidermis, suggesting a role in UVR-induced cancers. Obviously, more studies are needed to reveal whether CLCA2 indeed influences SCC initiation or progression and what are the mechanisms involved.

## Supplementary Information

Below is the link to the electronic supplementary material.

**Supplemental figure 1. Clca2** siRNA resulted in approx. 80% down-regulation of *Clca2* mRNA expression. The data represent means and range from two individual experiments, each performed with duplicate cultures (TIF 921 KB)

**Supplemental figure 2. **Effect of *Clca2* siRNA suppression on cell migration. siRNA silencing of *rClca2* did not significantly influence REK cell migration in a scratch wound assay. The data represent means and SE from five independent experiments. The statistical differences were tested using Pairwise *t* test (TIF 1205 KB)

** Supplemental figure 3. **Histological section from UVR-exposed mouse back skin stained with anti-CLCA2 antibody. Mild hyperplasia shows regular CLCA2 staining in the epidermis (TIF 6976 KB)

Supplementary file4 (DOCX 15 KB)

Supplementary file5 (DOCX 14 KB)

## Data Availability

The datasets generated during and/or analyzed during the current study are available from the corresponding author on reasonable request.
