# Supplementary figures and images for: The calcium-activated chloride channel-associated protein rCLCA2 is expressed throughout rat epidermis, facilitates apoptosis and is downmodulated by UVB
Source: Histochem Cell Biol. 2021 Jan 23;155(5):605–15. doi: 10.1007/s00418-021-01962-5 (PMC8134295; doi:10.1007/s00418-021-01962-5)

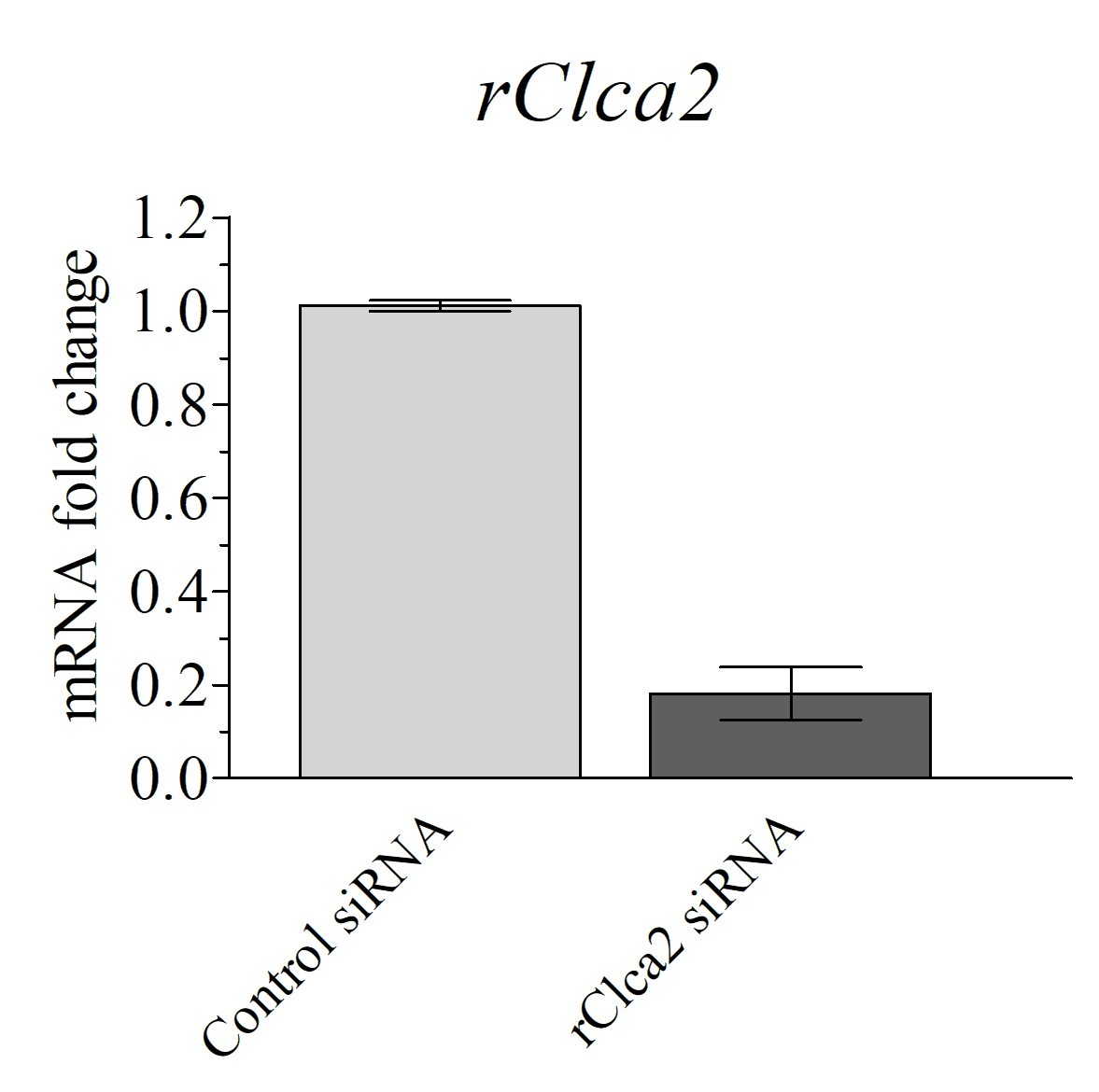

Supplement: Supplementary file 1 — Supplemental figure 1. Clca2 siRNA resulted in approx. 80% down-regulation of Clca2 mRNA expression. The data represent means and range from two individual experiments, each performed with duplicate cultures (TIF 921 KB) [file 418_2021_1962_MOESM1_ESM.tif]

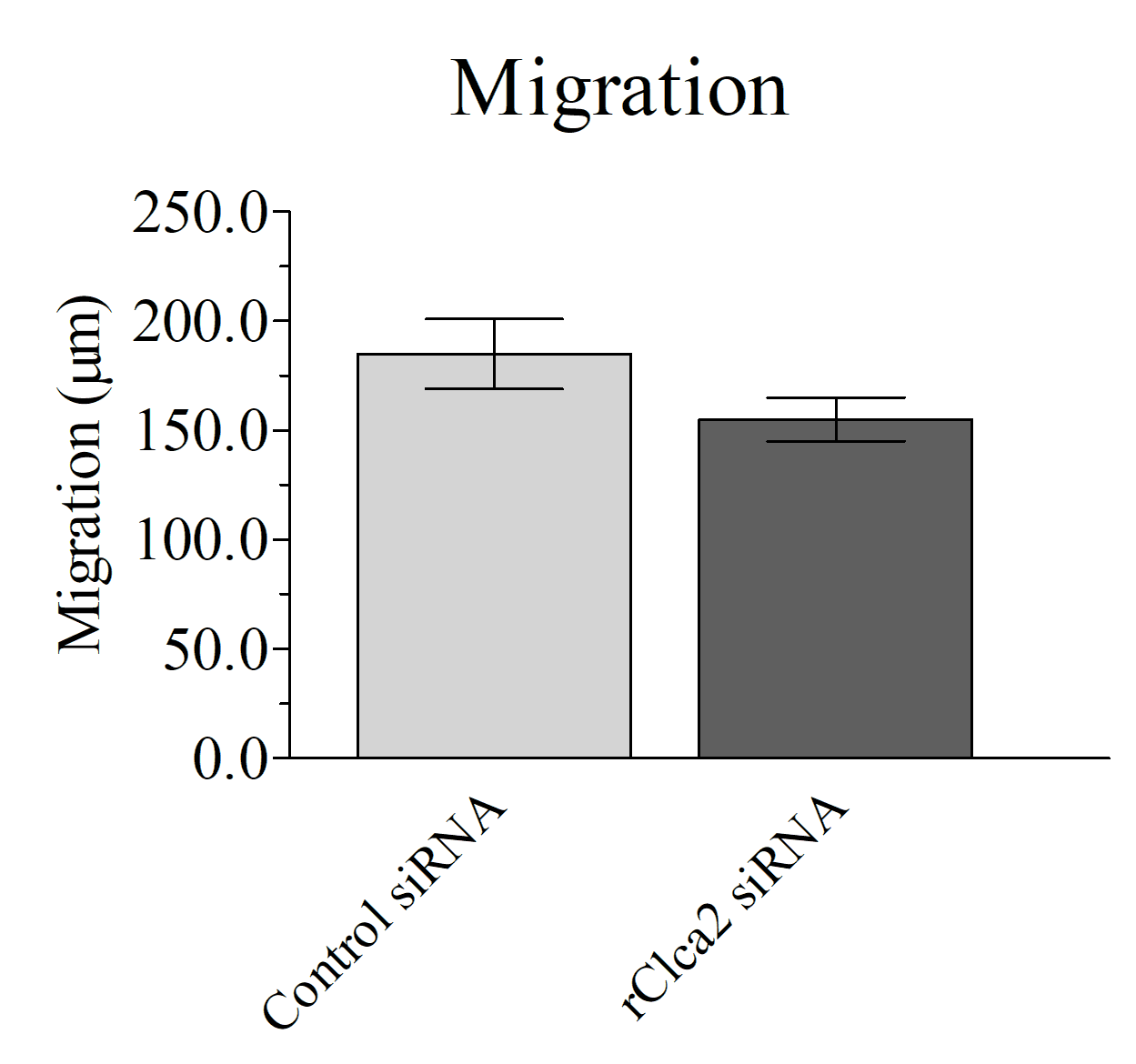

Supplement: Supplementary file 2 — Supplemental figure 2. Effect of Clca2 siRNA suppression on cell migration. siRNA silencing of rClca2 did not significantly influence REK cell migration in a scratch wound assay. The data represent means and SE from five independent experiments. The statistical differences were tested using Pairwise t test (TIF 1205 KB) [file 418_2021_1962_MOESM2_ESM.tif]

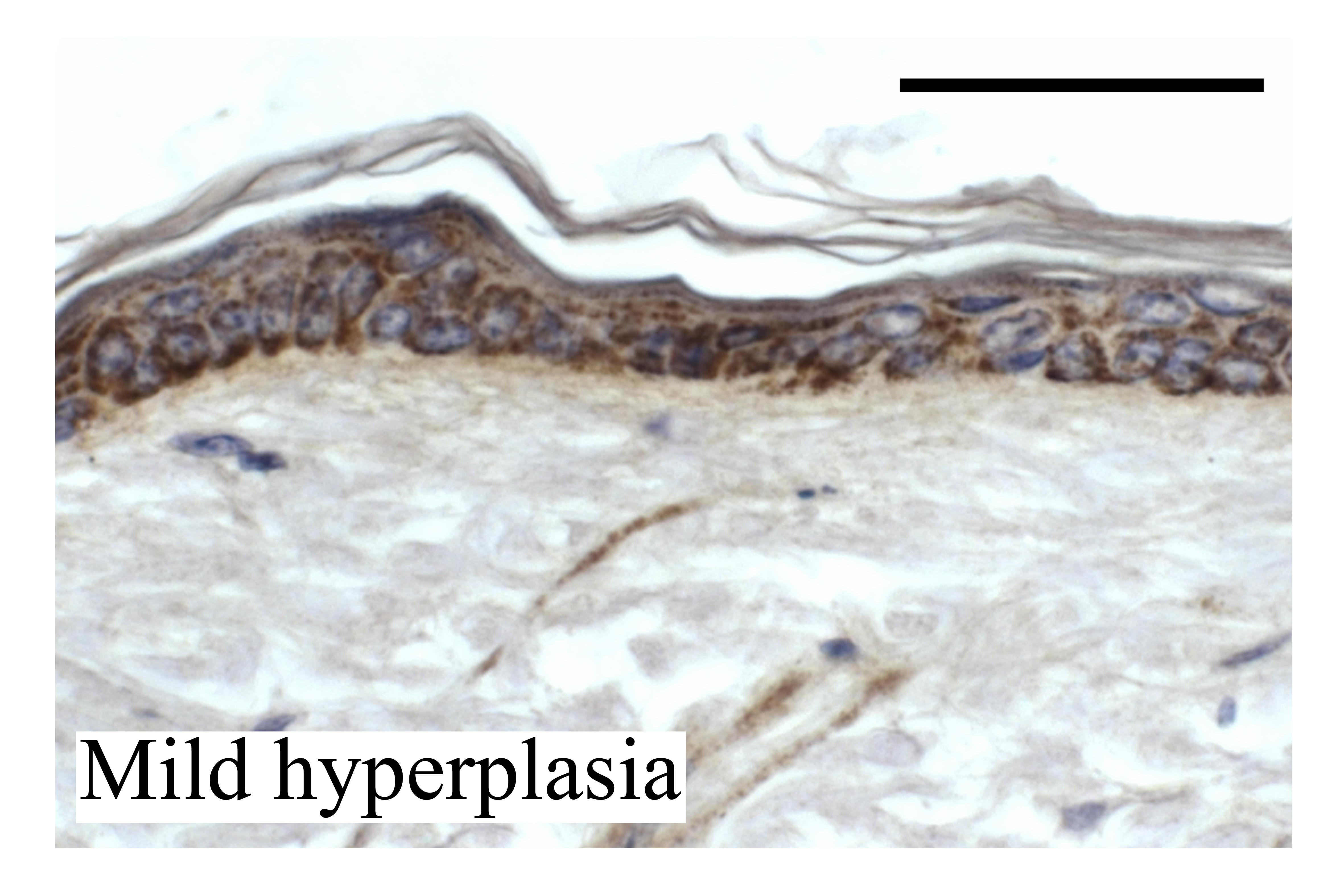

Supplement: Supplementary file 3 — Supplemental figure 3. Histological section from UVR-exposed mouse back skin stained with anti-CLCA2 antibody. Mild hyperplasia shows regular CLCA2 staining in the epidermis (TIF 6976 KB) [file 418_2021_1962_MOESM3_ESM.tif]
