# Supplementary material for: The calcium-activated chloride channel-associated protein rCLCA2 is expressed throughout rat epidermis, facilitates apoptosis and is downmodulated by UVB
Source: Histochem Cell Biol. 2021 Jan 23;155(5):605–15. doi: 10.1007/s00418-021-01962-5 (PMC8134295; doi:10.1007/s00418-021-01962-5)
Supplement: Supplementary file 4 — Supplementary file4 (DOCX 15 KB) [file 418_2021_1962_MOESM4_ESM.docx]

**Supplemental table 1.** The cycling conditions were as follows: preincubation for 15 min at 95°C, then 45 cycles of 20 s denaturation at 95°C, 20 s annealing at a primer-specific temperature and 20 s elongation at 72°C.

| **Gene** | **NCBI Reference Sequence:** | **Primer sequences (5’-3’)** | **Ann. T** | **Amplicon size** |
| --- | --- | --- | --- | --- |
| *Rplp0* | NM_ 022402.2 | Forward: GTGGTGATGCCCAAAGCTTG  Reverse: AGATGCAGCAGATCCGCAT | 60°C | 319 bp |
| *rClca2* | NM_001107450.1 | Forward: AGCATGGGCACTGGACTTAC  Reverse: TATAACATCGGCGCCTGCTC | 60°C | 287 bp |
