# Supplementary material for: The calcium-activated chloride channel-associated protein rCLCA2 is expressed throughout rat epidermis, facilitates apoptosis and is downmodulated by UVB
Source: Histochem Cell Biol. 2021 Jan 23;155(5):605–15. doi: 10.1007/s00418-021-01962-5 (PMC8134295; doi:10.1007/s00418-021-01962-5)
Supplement: Supplementary file 5 — Supplementary file5 (DOCX 14 KB) [file 418_2021_1962_MOESM5_ESM.docx]

**Supplemental table 2:** Presence of irregular CLCA2 staining in the epidermis and SCC.

Irregularity scores

____________________________________

n of samples 1 2 3 4

________________________________________________________________________________

Sham exposed specimens 14

Epidermis all areas 14 0 0 0

UVR exposed specimens 14

Epidermis all areas 4 0 6 4***

normal area 6 3 0 0

hyperplastic area 0 1 3 4^**^

dysplastic area 0 0 0 2

SCC 0 0 0 4^**^

The sections were scanned through using 40x objective and each consecutive field was scored for the morphological type (normal/mild hyperplasia, and hyperplasia, dysplasia) and presence of SCC. Each specimen contained one or more of these morphological types. Each 40x objective field was also evaluated for the CLCA2 staining pattern (regular or irregular containing focal weakly stained areas among the intensely stained cells). Irregular staining was classified in four level scoring from 1 to 4. Score 1 was given when 0-25% fields contained focally reduced staining, score 2, 3 and 4 when 26-50 %, 51-75% and 76-100 % of fields contained focally reduced staining, respectively. The scores are shown both per whole epidermal area in the specimen irrespective of the morphological type of the fields and per each morphological type separately. The values represent the numbers of specimens scored at each level. Mann Whitney U-test significance level when compared to the sham group:  ^**^p<0.01, ***p<0.001
